# Potential occurrence of Zika from subtropical to temperate Argentina considering the basic reproduction number (*R*_0_)

**DOI:** 10.26633/RPSP.2017.120

**Published:** 2017-09-29

**Authors:** Pablo Orellano, Darío Vezzani, Nancy Quaranta, Rodolfo Cionco, Julieta Reynoso, Oscar Salomon

**Affiliations:** 1 CONICET Universidad Tecnológica Nacional Argentina CONICET, Universidad Tecnológica Nacional, Argentina.; 2 CONICET Universidad Nacional del Centro de la Provincia de Buenos Aires Argentina CONICET, Universidad Nacional del Centro de la Provincia de Buenos Aires, Argentina.; 3 CIC Universidad Tecnológica Nacional Argentina CIC, Universidad Tecnológica Nacional, Argentina.; 4 Hospital Interzonal General de Agudos “San Felipe” Hospital Interzonal General de Agudos “San Felipe” Argentina Hospital Interzonal General de Agudos “San Felipe”, Argentina.; 5 CONICET Instituto Nacional de Medicina Tropical Argentina CONICET, Instituto Nacional de Medicina Tropical, Argentina.

**Keywords:** Zika virus infection, Aedes, disease vectors, epidemiologic models, Argentina, Infección por el virus Zika, Aedes, vectores de enfermedades, modelos epidemiológicos, Argentina, Infecção pelo Zika virus, Aedes, vetores de doenças, modelos epidemiológicos, Argentina

## Abstract

**Objective.:**

*To assess the potential occurrence of Zika transmission throughout Argentina by the mosquito Aedes aegypti considering the basic reproduction number (R_0_)*.

**Methods.:**

*A model originally developed for dengue was adapted for Zika. R_0_ was estimatedas a function of seven parameters, three of them were considered temperature-dependent. Seasonal Zika occurrence was evaluated in 9 locations representing different climatic suitability for the vector. Data of diary temperatures were extracted and included in the model. A threshold of R_0_ = 1 was fixed for Zika occurrence. Sensitivity analyses were performed to evaluate the uncertainty around the results*.

**Results.:**

*Zika transmission has the potential to occur in all studied locations at least in some moment of the year. In the northern region, transmission might be possible throughout the whole year or with an interruption in winter. The maximum R_0_ was estimated in 6.9, which means an average of 7 secondary cases from a primary case. The probabilistic sensitivity analysis showed that during winter the transmission can only be excluded in the southern fringe of geographic distribution of the vector and in part of central Argentina*.

**Conclusion.:**

*Zika virus has the potential to be transmitted in Argentina throughout the current geographic range of the mosquito vector. Although the transmission would be mainly seasonal, the possibility of winter transmission cannot be excluded in northern and central Argentina, meaning that there is a potential endemic maintenance of the disease*.

## RESUMEN

**Objetivo.:**

Evaluar la posibilidad de transmisión de zika en la Argentina por el mosquito *Aedes aegypti* considerando el número de reproducción básica (*R_0_*).

**Métodos.:**

Se adaptó para el zika un modelo desarrollado originalmente para el dengue. Se estimó *R_0_* en función de siete parámetros, tres de los cuales se consideraron dependientes de la temperatura. Se evaluó la ocurrencia estacional de zika en nueve localidades que representan diferente aptitud climática para el vector. Se obtuvieron los datos de las temperaturas diarias y se incluyeron en el modelo. Se fijó un umbral de *R_0_* = 1 para la ocurrencia de zika. Se realizaron análisis de sensibilidad para evaluar la incertidumbre de los resultados.

**Resultados.:**

En todos los lugares estudiados es posible la transmisión de zika al menos en algún momento del año. En la región norte, la transmisión podría ser posible durante todo el año o con una interrupción en invierno. Se estimó el *R_0_* máximo en 6,9, lo que significa un promedio de siete casos secundarios a partir de un caso primario. El análisis de sensibilidad probabilística demostró que durante el invierno la transmisión sólo puede ser excluida en la franja sur de distribución geográfica del vector y en parte de la región central de Argentina.

**Conclusión.:**

El virus del Zika puede ser transmitido en Argentina en todo el rango geográfico actual del mosquito vector. Aunque la transmisión sería principalmente estacional, no es posible descartar la posibilidad de transmisión invernal en el norte y centro de la Argentina, lo que significa que la enfermedad puede mantenerse de manera endémica.

## RESUMO

**Objetivo.:**

Avaliar a ocorrência potencial de transmissão de zika em Argentina pelo mosquito *Aedes aegypti* considerando o número de reprodução básico (*R_0_*).

**Métodos.:**

Foi adaptado para zika um modelo originalmente desenvolvido para dengue. *R_0_* foi estimado como uma função de sete parâmetros, três deles considerados dependentes da temperatura. A ocorrência de zika sazonal foi avaliada em nove locais que representam diferentes adequações climáticas para o vetor. Os dados das tempera-turas diárias foram extraídos e incluídos no modelo. Um limite de *R_0_* = 1 foi fixado para a ocorrência de zika, e foram realizadas análises de sensibilidade para avaliar a incerteza em torno dos resultados.

**Resultados.:**

A transmissão de zika pode ocorrer em todos os locais estudados pelo menos em algum momento do ano. Na região norte, a transmissão pode ser possível durante todo o ano ou com uma interrupção no inverno. O *R_0_* máximo foi estimado em 6.9, o que significa uma média de 7 casos secundários a partir de um caso primário. A análise de sensibilidade probabilística mostrou que durante o inverno a transmissão só pode ser excluída na franja sul da distribuição geográfica do vetor e em parte da Argentina central.

**Conclusão.:**

O vírus Zika tem o potencial de ser transmitido na Argentina ao longo da atual faixa geográfica do vetor. Embora a transmissão seja principalmente sazonal, a possibilidade de transmissão no inverno não pode ser excluída no norte e centro da Argentina, o que significa que existe uma potencial manutenção endêmica da doença.

A bulk of viruses transmitted by mosquitoes are emerging or reemerging globally as consequence of global warming, urbanization and modern transport networks. Among these viruses, Zika was first isolated in a forest area in Uganda, Africa (1), and was historically restricted to Asia and Africa for more than 50 years (2), but recently spread to Micronesia in 2007and Brazil in 2014–2015 (3,4), continuing its expansion through South, Central and North America (2). By 2016, more than 45 countries and territories throughout the Americas have reported more than 577 000 locally-acquired cases, with estimates of several million real cases (2), and 165 932 cases reported only in Brazil (5). The potential burden associated with this disease is still to be determined, but reports of Guillain-Barre syndrome and other neurologic complications in adults (6), in addition to microcephaly and other serious brain abnormalities in newborns (5), have positioned the Zika infections as a severe threat to public health. The high proportion of asymptomatic patients (7) makes difficult the rapid detection of the autochthonous transmission, facilitating the spread of this disease. In addition, the main vectors of Zika have currently a broad range of global distribution, meaning that a large portion of the world population is at risk of this arbovirus infection.

Although Zika virus was detected in semen with high viral load (8), and sexual and donor transmission has been documented (9), the main transmission route is through vector bite (10). The primary vector is *Aedes aegypti*, while *Ae. albopictus* has proved to be less competent (11,12) and the common mosquitoes *Culex pipiens* and *Cx. quinquefasciatus* were discarded as Zika vector (13,14). Therefore, the geographic distribution of *Ae. aegypti* determines *a priori* the risk of epidemics. Under the current disease expansion, epidemiologic models could be useful tools to assess the potential boundaries of different risk areas, the environmental suitability for seasonal and endemic transmission cycles, and the potential future burden of the Zika infections. In addition, simple models that can be applied using spreadsheets and available meteorological data may help national and local epidemiologists to evaluate the transmission risks and the course of future interventions.

Argentina exhibits the boundary of vector’s southern distribution in the Americas. Considering the current vector distribution, the epidemiologic Zika situation in South America, and the recent first outbreak in the country (15), it is imperative to assess the potential occurrence of Zika transmission throughout Argentina by the mosquito *Ae. aegypti*. With this aim, a simple model considering the basic reproduction number based on daily ambient temperatures was used to evaluate the potential transmission in different regions representing a wide range of temperatures.

**TABLE 1.** Geographic location and potential risk of virus transmission of the selected meteorological stations

Potential risk	Province	Station	Nearest city	Latitude and longitude	Altitude a.s.l. (meters)
High	Misiones	S1	Puerto Iguazú	-25.62, -54.67	261
Salta	S2	Saucelito	-23.47, -64.38	350
Tucumán	S3	Famaillá	-27.02, -65.38	380
Medium	Córdoba	S4	Manfredi	-31.86, -63.75	292
Buenos Aires (North)	S5	Ituzaingó	-34.61, -58.67	22
Entre Ríos	S6	Concepción del Uruguay	-32.49, -58.35	17
Low	Buenos Aires (South)	S7	Las Armas	-37.09, -57.88	28
La Pampa	S8	Anguil	-36.54, -63.99	165
Río Negro	S9	Barda del Medio	-38.74, -68.11	297

***Source:***Prepared by the authors.

## MATERIALS AND METHODS

### Settings

The study area comprised the current known distribution of *Ae. aegypti* in Argentina. Three sub-areas were defined according to autochthonous dengue records as a proxy for the risk of virus transmission by *Ae. aegypti*: 1) a high risk area in the northeast and northwest of the country (latitude above -28.00°), where outbreaks occurs almost annually; 2) a medium risk area in central Argentina (between -28.00° and -35.00°), where large and small outbreaks occur sporadically; and 3) a low risk area in the southern distribution limit of the vector (latitude below -35.00°), where autochthonous dengue transmission was never recorded or only a few isolated cases were notified. Within each area, three meteorological stations were selected (Table 1). The station from Tucumán province (S3) was specifically included because its proximity to San Miguel de Tucumán, where the first outbreak of Zika in the country was recently confirmed with 25 autochthonous cases (15).

Daily temperatures were obtained from the automatic meteorological stations net of the Instituto Nacional de Tecnología Agraria (INTA) (16). Records from years 2012 to 2016 were used, and daily means were calculated to obtain a representative dynamic of mean ambient temperatures throughout a typical recent year.

### Model Overview

The basic reproduction number (*R_0_*) was used to estimate the potential of *Ae. aegypti* to transmit Zika virus through a wide range of temperatures in Argentina. For a vector-borne disease, *R_0_* is defined as the number of persons who would be infected from a single person initially infected by a mosquito (17). Following the model developed by Liu-Helmersson et al. for the potential transmission of dengue (18,19), the *R_0_* was calculated using the vectorial capacity (*Vc*) and the duration of the infected period (*Th*) [equation 1].

$$R_{0}=T_{h}V_{c}[1]$$

Similar to the *R_0_*, the *Vc* is defined as the number of secondary cases of the disease generated by a primary case, but on a daily basis. As in the previously mentioned model, the *Vc* was estimated using an equation of six parameters. These parameters represent the vector biting rate, the human-to-vector infection, the vector mortality rate, vector-to-human population ratio, the probability of vector-to-human transmission, and the duration of the extrinsic incubation period. Only the first 3 parameters were considered temperature dependent. The outcome variable (*R_0_*) was calculated on a daily basis for each locality. A selection criterion was assumed to estimate the daily potential occurrence of Zika transmission, allowing the assessment of seasonal patterns.

### Outcome variable, input parameters and criteria

*Vc* was estimated through the following equation [2]:

$$V_{c}=ma^{2}b_{h}b_{m}\exp\left(-\mu_{m}n\right)/\mu_{m}[2]$$

where *m* represents the vector-to-hu-man population ratio, *a* is the average daily vector biting rate, *b_h_* is the probabil-ity of vector-to-human transmission perbite, *b_m_* is the probability of human-to-vector infection per bite, *µ_m_* is thevector mortality rate, and *n* is the dura-tion of the extrinsic incubation period.Equations [1] and [2] can be combined to obtain a single equation for the *R_0_* [Equa-tion 3], including the duration of the infectious period (*T_h_*):

$$R_{0}=T_{h}ma^{2}b_{h}b_{m}\exp\left(-\mu_{m}n\right)/\mu_{m}[3]$$

Parameters *a* and *µ_m_* were not modified from the original model because they are specific for *Ae. aegypti*, the vector for both dengue and Zika viruses in America. The value of *b_h_* was previously estimated for different flavivirus, including West Nile virus, Murray Valley encephalitis virus, and St. Louis encephalitis virus (20). Accordingly, we have used the same un-modified equations. The vector-to-human population ratio (*m*) was assumed to be 1.5, as in the referenced paper (19), because in this model we are searching for a threshold value for Zika transmission, and accordingly the maximum potential transmission was assumed independent from other variables. More detailed models allow reduction of this parameter, i.e. by reducing the vector density through larval control measures. The extrinsic incubation period (*n*) and the probability of human to vector infection per bite (*b_m_*) were estimated according to data from laboratory experiments with*Ae. aegypti* and Zika virus (21,22). The duration of the infectious period (*T_h_*) was assumed to be 5 days (23). These four parameters were considered independent from the temperature effect. All parameters and values can be seen in Table 2.

A threshold value of *R_0_* = 1 was fixed for the occurrence of Zika transmission. If the value of the *R_0_* was above this cut-off point, the transmission was assumed as possible in a certain day of the year (24).

### Sensitivity analysis

We performed deterministic and probabilistic sensitivity analyses based on five parameters that contributed with uncertainty to the model, i.e. temperature, *m*, *b_m_*, *n* and *T_h_*. Two-way deterministic sensitivity analyses was used to evaluate the variation of the *R_0_* in a range of +/20% around central values of *b_m_* and *n*, in a range from 1 to 2 for *m*, in a range from 1 to 10 days for *T_h_*, and finally in a range of temperatures from 20°C to 30°C.

**TABLE 2.** Equations and references for model parameters

Parameter	Value	References
Vector-to-human population ratio (*m*)	= 1.5	(19)
Average daily vector biting rate (*a*) (1/day)	= 0.0043 *T*_m_ + 0.0943	(18)
Probability of human-to-vector infection per bite (*b*_m_)	= 0.62	(22)
Probability of vector-to-human transmission per bite (*b*_h_)	= 0.001044 *T*_m_ (*T*_m_ 12.286) √(32.461 *T*_m_) for (12.286°C<*T*_m_<32.461°C)	(18,20)
= 0 for (*T*_m_ < 12.286°C)	
= 1 for (32.461°C<*T*_m_)	
Duration of the extrinsic incubation period in days (*n*)	= 10	(21,22)
Vector mortality rate (*μ*_m_)	= 0.8692 0.159 *T*_m_ + 0.01116 *T*_m_^2^ -0.0003408 *T*_m_^3^ + 0.000003809 *T*_m_^4^ for (10.54°C<*T*_m_<33.41°C)	(18,25)
		
= 1 for (*T*_m_ < 10.54°C) or (33.41°C < *T*_m_)		
Duration of the infectious period in days (*T*_h_)	= 5	(23)

***Source:*** Prepared by the authors. ***T*_m_:** Mean daily temperature

A probabilistic sensitivity analysis was also performed through the Monte Carlo method, that allowed the simultaneous variation of *m*, *b_m_*, *n*, *T_h_* and temperature within the previously considered ranges. The mean and standard deviation of the temperature were calculated for each me-teorological station during January and June, the warmest and the coldest months, respectively. The *m*, *b_m_*, *n*, *T_h_* were assumed to follow a triangular distribution, because little is known about the real distribution and boundaries of these parameters. The temperature was assumed to follow a normal distribution. This variable also influenced to *a*, *b_h_* and *µ_m_*, which means that all parameters within the model were subject to variations. However, the variation of parameters *m*, *b_m_*, *n*, *T_h_* accounted for the lack of knowledge around them (also called epistemic uncertainty), while the variation in the temperature accounted for the natural variability (also called aleatory uncertainty) in each station and month (26). Accordingly, this sensitivity analysis only took into account the lack of knowledge around the specific Zika transmission parameters, and not around the *Ae. aegypti* and general flavivirus parameters. For each station, 10 000 simulations were run and the 95% CI of the *R_0_* during the warmest and coldest months were calculated assuming a normal distribution of the outcome variable.

All calculations, sensitivity analyses and graphics were performed using the “lattice” package (27,28) and the “mc2d” package (29) in the statistical software R, version 3.2.2 (https://www.r-project.org/).

## RESULTS

### *R_0_* dynamics

**FIGURE 1.**
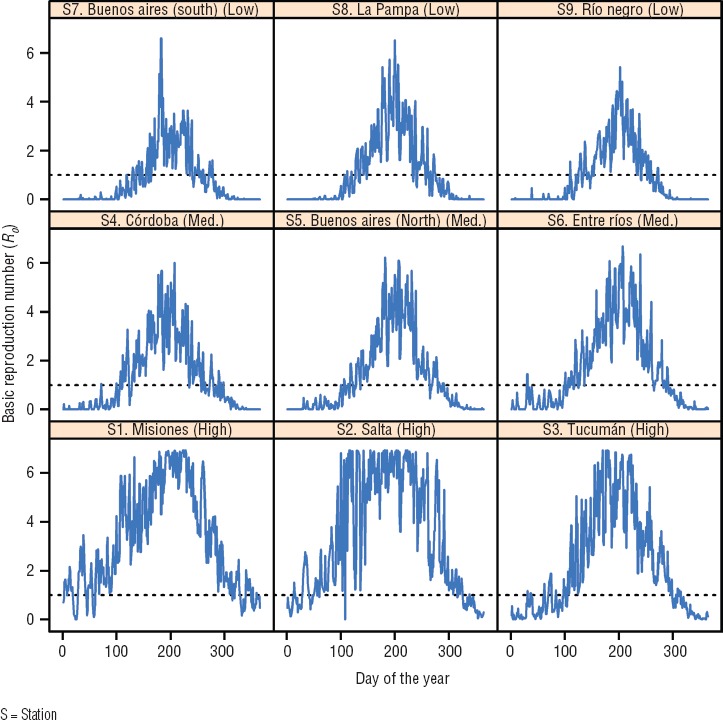
Potential transmission of Zika in 9 locations, Argentina

According to the model, Zika transmission has the potential to occur throughout all climatic regions of the country where *Ae. aegypti* is present, at least in some moment of the year (Figure 1). The highest values of *R_0_* were observed in high risk areas (bottom plots), whereas a shorter period could be identified in medium risk areas (middle plots), and finally, a more variable *R_0_* dynamics was restricted to summer period in low risk areas (upper plots).

In the northern region of the country, two different scenarios were identified. One with transmission throughout the whole year (Misiones province), and other with the interruption in winter during one or three months (Salta and Tucumán province, respectively). In these settings, the maximum *R_0_* would be 6.9,which means an average of 7 secondary cases from a primary case. The maximum number of potential transmission days was estimated to be 316 for Misiones province. In central Argentina, the potential transmission was restricted to October-April (Córdoba and northern Buenos Aires), or July-April (Entre Ríos), but always with several interruptions, i.e. days with *R_0_* < 1. The maximum number of potential transmission days was estimated in 173 for Entre Ríos province, meaning that Zika transmission may occur only during the half part of the year. However, the maximum *R_0_* observed in the region (i.e. 6.7 in Entre Ríos) was next to those of the Northern provinces. Finally, in the Southern distribution limit of the vector, the maximum *R_0_* was estimated to be 6.6 (southern Buenos Aires), but nevertheless Zika transmission can occur only during a maximum of 132 days (La Pampa), and restricted to spring and summer months.

### Sensitivity analyses

**FIGURE 2.**
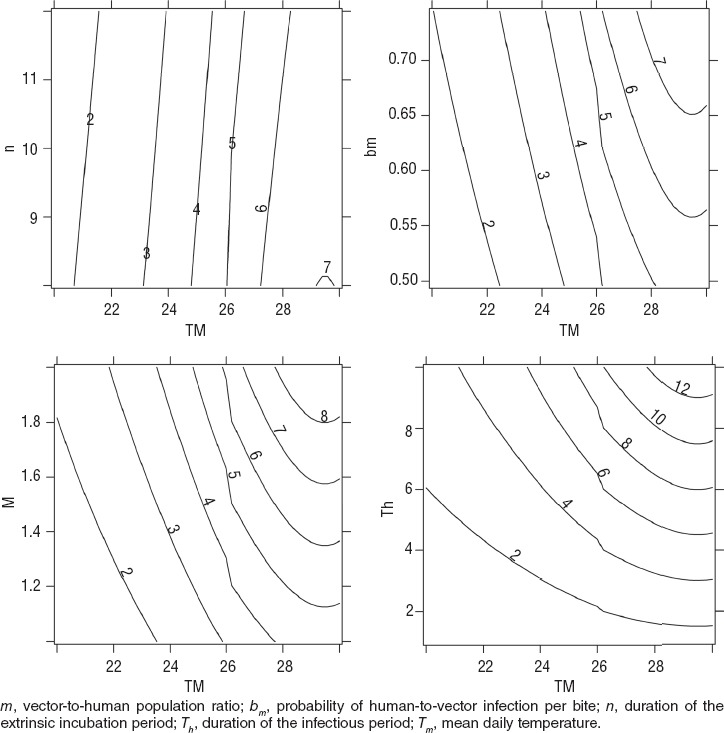
Two-way deterministic sensitivity analysis

According to the two-way deterministic sensitivity analysis, all parameters showed a value of *R_0_* > 1 within the temperature range of 20–30°C, thereby allowing for Zika transmission (Figure 2). However, *n*, *b_m_* and *m* showed weak influence on the *R_0_* below 28°C. By contrast, *T_h_* showed a strong influence on *R_0_* within the whole range evaluated.

The probabilistic sensitivity analysis (Table 3) showed that during the warmest month Misiones province can be considered of significant potential for Zika transmission, due to the exclusion of the 1 value from its confidence interval. In all other stations from northern, central and southern regions the 1 threshold is included within the confidence interval of the R_0_,and thus the Zika potential transmission cannot be either confirmed or ruled out. During the coldest month, the transmission of Zika cannot be excluded in Entre Ríos and in northern Argentina. On contrary, the transmission can be excluded in the three stations encompassing the southern fringe of geographic distribution of the vector and in two out of three stations from central Argentina (Córdoba and north Buenos Aires), given that the upper values of the *R_0_* confidence interval are below 1.

## DISCUSSION

Our assessment suggests that the Zika virus has the potential to be transmitted in Argentina throughout the entire geographic range of the mosquito *Ae. aegypti*. Although the transmission would be mainly seasonal, the possibility of winter transmission cannot be excluded in northern and central Argentina, meaning that there is a potential endemic maintenance of the disease. In this sense, the first cases of dengue transmission during winter were recently reported in the Northeast of the country (15), since the first dengue outbreaks in 1998 (30). Considering the similarities of both *Aedes*-borne diseases, it could be expected a similar spread pattern. On the other hand, the Zika virus has proved to be capable of being vertically transmitted in *Ae. aegypti* (31), allowing the survival of the virus in adverse conditions, and thus increasing the probability of an endemic cycle in these latitudes. Globally, an increasing number of data is showing that Zika virus transmission by *Ae. aegypti* mosquitoes would be endemic in tropical, subtropical and even temperate latitudes (32,33).

Our model, based on that of Liu-Helmersson et al. (18,19), suggests a Zika transmission boundary up to the latitude of 39° south. The same model adapted by Rocklov et al. for Zika in Europe and Asia (34) identified the potential spread of the virus during summer up to the lat-itude of nearly 50° north. But it is worthnoting that our estimations were restricted to the southern distribution limits of Ae. aegypti in America, and therefore, it is probably a Zika spreading to the south if vector distribution increase. Another model performed to predict global Zika spread (35) match with our estimations for northern and central Argentina. However, that model used land cover, human population density and other non-meteorological factors, in addition to ambient temperature. Remarkably, when the mentioned model only considers environmental factors, Zika occurs up to Tierra del Fuego, a very cold location far away from the current distribution of *Aedes* vectors. For Africa and Asia, another model (36) projected the potential for Zika transmission considering dengue parameters, data of vector presence, and estimations of possible travelers from the Americas within the viremic period. The geographical area of Zika spread was estimated to reach the latitude of 34° south, coincident with the region of central Argentina classified as medium risk in our study.

Regarding *R_0_* calculation, theoretical models have estimated central values in the range of 2.1 – 4.8 for *Ae. aegypti* and *Ae. albopictus* (34,37–39). Empirical estimations based on the epidemics from Colombia in several cities yielded a wider range of 1.4 – 6.6 (40–42). The unique study using Big Data analysis to estimate the *R_0_* found a value of 2.6, within the range of both theoretical and empirical approaches (42). It should be observed that theoretical and semi-empirical models were parameterized for the use in America, Africa and Europe for both vector species, and the poor ability of *Ae. albopictus* as Zika vector was already established in several studies (12,43). This could partially explain the higher values of *R_0_* estimated in our study. The maximum value of *R_0_* = 6.6 obtained by Nishiura and colleagues in Colombia (41) was similar to our maximum value of *R_0_* in northern and central Argentina. Moreover, when considering the uncertainty around maximum likelihood estimates in their model, the maximum *R_0_* was estimated to be 14.8, similar to our *R_0_* for Misiones during January (i.e. 11.4). This consistence with the observed values could be useful as external validation for our model. Another undesired opportunity to validate our results was the recent Zika outbreak in Tucumán province (15). This outbreak, the first in the country, with 25 confirmed cases was small, but occurred during May, almost at the end of the potential period calculated. *R_0_* estimations from transmission dynamic models have suggested that Zika epidemics is not containable, large-scale outbreaks will occur with an interval of years, and the virus will eventually become endemic in Latin America (44).

**TABLE 3.** Temperatures registered during the warmest and coldest months (January and June, respectively), and results of the probabilistic sensitivity analysis for the basic reproduction number (*R*_0_); 95% confidence intervals were estimated through 10000 Monte Carlo simulations

Province	Station	Recorded period	Temperature in January (°C) (Mean, SD^a^)	Temperature in June (°C) (Mean, SD^a^)	*R*_0_ in January(95% CI)	*R*_0_ in June(95% CI)
Misiones	S1	2013–2016	28.26 (1.72)	18.61 (3.07)	1.2–11.4	0.0–3.8
Salta	S2	2013–2016	28.14 (2.51)	16.57 (2.89)	0.5–11.0	0.0–2.6
Tucumán	S3	2012–2016	26.64 (2.66)	14.12 (2.73)	0.1–10.3	0.0–1.5
Córdoba	S4	2012–2016	24.64 (2.76)	10.99 (3.54)	0.0–8.6	0.0–0.85
Buenos Aires (North)	S5	2012–2016	25.13 (3.11)	11.44 (3.50)	0.0–9.1	0.0–0.9
Entre Ríos	S6	2012–2016	25.69 (2.91)	12.36 (3.83)	0.0–9.5	0.0–1.36
Buenos Aires (South)	S7	2012–2016	22.81 (3.58)	9.8 (3.03)	0.0–7.6	0.0–0.4
La Pampa	S8	2012–2016	24.71 (3.74)	9.13 (3.29)	0.0–9.1	0.0–0.4
Río Negro	S9	2012–2016	24.34 (3.06)	8.68 (3.60)	0.0–8.6	0.0–0.4

^a^ SD: standard deviation. ***Source:***Prepared by the authors based on the study results.

Sensitivity analysis showed a strong dependence of *R_0_* on the ambient temperature, whereas all others parameters demonstrated a weak association. Only for temperatures above 28°C, the parameters *n*, *b_m_* and *m* had a moderate influence on *R_0_*. On contrary, *T_h_* showed a stronger influence on *R_0_* in the whole range of temperatures. This means that within the considered ranges, parameters associated with the cycle of the virus in the mosquito, the susceptibility to Zika virus and the vector-to-human population ratio have less importance than the duration of the infectious period. These results may have consequences regarding the control measures, given that the dynamic of the disease depends more on intrinsic host parameters and environment temperatures than on mosquito densities. A similar dependence of *R_0_* on temperature and diurnal temperature range was previously described by Liu-Helmersson et al. (18) in the original model for dengue. In this sense, our study used observed daily temperature data instead of projections, and thus the diurnal variation of temperatures has been implicit in calculations. The high dependency of *R_0_* on ambient temperatures highlights the fact that Zika transmission would have a seasonal behavior in subtropical and temperate Argentina, due to the marked difference of temperature between summer and winter. In tropical settings like Central America, the Zika virus might show a different transmission dynamic, with no seasonality or a stronger dependence on other environmental factor like the precipitation.

Our approach was subject to some limitations. First, research on Zika susceptibility by mosquitoes and humans are ongoing, and thus our parameters may be modified as more studies are published. Even though our sensitivity analyses showed a weak dependence of *R_0_* on these parameters, future research could change the main results. Other parameters regarding the transmission dynamic also need to be confirmed, particularly, the duration of the infectious period in humans has demonstrate a strong influence on the *R_0_*. Second, a significant amount of uncertainty was considered for all these parameters, and this was reflected in the wide range of the confidence intervals around the *R_0_* obtained by the probabilistic sensitivity analysis. Finally, our estimations of the *R_0_* are based almost exclusively on ambient temperature, while the vector-to-human population ratio was fixed and actions to control vector populations were not considered. In this regard, this model should be visualized as a tool to understand the climatic favorability for the seasonal or endemic transmission of the disease, and not as a model to analyze other environmental, anthropic or interventional influences on the transmission.

Further development is needed to serve as a tool for the analysis of different strategies for public health interventions, e.g. larval control interventions and fumigations. However, interventions related to human behavior (both vector control and prevention) should be similar to those for dengue, excluding the sexual transmission that, as previously stated, seems not to play a major role in the transmission dynamic. Other important weather variable that was not considered in our model was precipitation. This variable has a major influence on water availability in artificial containers used by *Ae. aegypti* as breeding sites, affecting mosquito densities. As we have previously stated, our model was aimed to predict climatic favorability and not mosquito density, which was assumed to be sufficient for the maximum potential transmission, independently of other variables. More detailed models, as the DENSiM developed by Focks et al. for dengue (45), consider mosquito density by person and other variables, and goes beyond only weather variables as predictors. In the case of Zika, more information is needed about the biology of the virus to develop more complex models.

In brief, we used a simple temperature-dependent model developed for dengue (18), and by means of changing a number of parameters we were able to apply the same procedure for the estimation of the favorability for the Zika virus circulation in Argentina, the risk of seasonal transmission, and the possibility of the endemic establishment in the region. This simple procedure can be reproduced by public health professionals and health decision makers to evaluate the risk of Zika transmission at a national or local level.

### Disclaimer.

Authors hold sole responsibility for the views expressed in the manuscript, which may not necessarily reflect the opinion or policy of the *RPSP/PAJPH* or PAHO.
